# Living in an Age-Friendly Community: Evidence from a Masterplanned Development in Southwest Sydney

**DOI:** 10.3390/ijerph20021312

**Published:** 2023-01-11

**Authors:** Alasdair Jones, Susan Parham

**Affiliations:** 1 Human Geography, Department of Geography, University of Exeter, Exeter EX4 4RJ, UK; 2 Urbanism and Planning, University of Hertfordshire, Hatfield AL10 9EU, UK

**Keywords:** age-friendly communities, built environment, urban planning, masterplanning, aging in place, qualitative research, residential environments

## Abstract

This paper is situated at the interface of a growing urban studies literature concerned with ‘masterplanning’ practices in urban planning and another, hitherto relatively discrete, body of research concerned with age-friendly cities and communities. The authors are interested in exploring a gap in aging in place literature around how neighbourhoods and residential settings developed with aging in place principles in mind are experienced and perceived by residents. To explore this research gap, the authors analyse qualitative (primarily interview and focus group) data collected in Park Central, a masterplanned development located in the Campbelltown suburb in the southwest region of the Sydney metropolitan area, Australia. This development was delivered in response to a need identified by the state’s land and property development agency, Landcom, for more diverse and affordable medium-density housing in Campbelltown. In particular, a need was identified for housing developments that would be able to sustainably accommodate the changing lifestyle needs of a maturing population in the region. Drawing on our thematic analysis of our data, we discern three key themes in research participants’ experiences of Park Central as a place for aging. These themes are elaborated via recourse to excerpts from our data and discussed with a view to informing how the conception, development and practice of further age-friendly communities is approached.

## 1. Introduction

As our understanding of preferences for, and inhibitors to, ‘aging in place’ (AIP) has grown, important gaps in our knowledge have been identified. Critically to this paper, these gaps include an experiential gap, or a gap in our understanding of how people aging in places developed with principles of AIP in mind experience that process. As Lewis and Buffell put it, “to date, there has been limited research focusing on the places of aging, and how these affect the experience of aging in place” (p. 1, [1]). This paper seeks to contribute to filling this gap by presenting qualitative evidence focused on experiences of AIP from an Australian case study neighbourhood characterized by features associated with ‘age-friendly communities’ [2,3,4,5,6,7].

Specifically, in this paper we use qualitative (interview and focus group) data collected with older people living in a masterplanned development in the southwestern Sydney metropolitan area to explore not only how those residents experienced the development as age-friendly, but also how they co-produced it as a ‘place for aging’ in various ways. Through our analysis, we find that perceptions and practices of our case study masterplanned development as ‘age-friendly’ were not only a function of the planning principles that informed, and urban design features of, the development, but also of the ways that the development was (at least in its early stages) managed and of the community development activities of the older residents themselves.

In the growing academic and policy literature on AIP, a number of approaches and principles for facilitating and enabling age-friendly residential areas have been identified [8,9,10]. Building on these conceptual developments, various models of AIP—such as naturally occurring retirement communities (NORC-SSPs), villages and campus-affiliated communities [11]—have been specified. At the same time, there is a growing literature concerned with: housing preferences for AIP [12,13]; “how older people understand the meaning of ‘aging in place’” [14]; methodologies for auditing the built environment in relation to AIP [15] and for measuring the ‘age-friendliness’ of settings [16] (pp. 15,16) and [17]; studying environmental and psychosocial interventions aimed at promoting active ageing and fostering age-friendly cities [18]; the relationship between the built environment and mobility for older adults AIP [19,20]; and the relationship between the community environment and health for older adults [21].

As Lewis and Buffel recently observe, however, in this wider body of work on AIP and age-friendly communities, there is a lack of research concerned with how built environments are experienced as places for aging [1]. While a growing number of studies in a variety of national contexts [1,22,23,24,25] have been conducted to help fill this gap, notably these studies have tended to focus on how older adults perceive and experience the ‘age-friendliness’ [24] (among other things) of extant neighbourhoods that were not developed according to AIP and age-friendly community principles. Thus, as more and more neighbourhoods and residential settings have been developed and/or adapted in line with principles of age-friendly communities and AIP, it is evident that there is a shortage of research on how those sorts of settings are experienced as places for aging by residents.

Notably, this lack of evidence can be seen as part of a broader set of “gaps in our understanding of …[age-friendly community] initiatives’ development, implementation, sustainability, and effectiveness” (p. 178, [26]). These interrelated research gaps are incrementally being filled—e.g., with the development of an evaluation tool [27] and logic model [28] for evaluating the impacts and outcomes of age-friendly communities—and the present analysis can be seen as part of this wider work to understand and evaluate the (re)development of neighbourhoods and residential settings through urban planning and design approaches informed by AIP principles.

Moreover, this observed lack of evidence of how people experience the age-friendliness of built environments has synergies with a wider critical literature on AIP discussions [29,30] which is partly premised on the contention that AIP is “a term widely used in aging policy and research but underexplored with older people themselves” [14] (p. 357) and [31]). Such critiques have therefore sought to engage much more with naturalistic and in-depth understandings of the lived experience and practices of AIP. They include, for instance, extensive ethnographic studies that have explored AIP as a socially stratified process [32,33], mixed-methods qualitative research concerned with how the benefits of age-friendly environments are distributed inequitably [34], and resilience-oriented research concerned with older people’s capability to achieve the ‘functionings’ they value in regard to healthy ageing [35].

Elsewhere, others critical of AIP research to date have argued that there is a need to move beyond understanding older people’s experiences and practices of AIP, to more participatory research approaches oriented toward the co-design of age-friendly communities. Thus, case studies of age-friendly city development practices practice are providing valuable insights into the value of participation in decision making about housing, services, environments and mobility [36]. In Manchester, for example, older people are involved in developing holistic perspectives on lifetime neighbourhoods drawing on international good practice through participation in the WHO Global Network on Age Friendly Cities [36]. The development of co-design approaches is increasing the agency of older people themselves in determining how to successfully implement improvements to places’ age-friendliness, as recent evidence from three urban settlements in the UK makes clear [37].

While these are important critiques, it continues to be the case that naturalistic understandings of how older adults experience the built environment from an aging perspective are underdeveloped. Within this broader research gap, then, while a significant volume of research into experiences of living in retirement villages across various national contexts has been conducted (e.g., [38,39,40,41]), there is a narrower (but important) gap in our understanding of how purposefully designed and ‘masterplanned’ [42] age-friendly neighbourhoods are experienced (p. 16, [16]). While some in-depth findings are emerging around experiences of age-friendly urbanism [43], these are at the city scale and concerned specifically with the intersection of ‘smart-city’ technologies and the age-friendly agenda. This paper seeks to supplement this emerging literature, therefore, by honing in on this particular empirical research gap and presenting qualitative *neighbourhood-level* evidence about experiences of aging in a purposefully designed ‘place of aging’ located in southwest Sydney, Australia. This evidence was collected as part of a wider study of sustainable living practices in masterplanned developments in Australia and the UK.

## 2. Materials and Methods

The findings discussed in this paper derive from a much wider comparative study of sustainable living practices in five case study masterplanned developments in Australia (the Sydney Metropolitan Region) and the UK (southeast England). In that wider study (which is reported on in detail elsewhere [44,45,46]), the research team were interested in exploring, through the prisms of sustainable urbanism [47,48] and sustainable living practices [49,50], the interplay between urban design, masterplanning implementation and the everyday social practices of masterplanned developments.

As with the other studies of older adults’ experiences of urban neighbourhoods discussed in the preceding review [1,22,23], methodologically the wider ‘sustainable living practices’ study from which the present paper derives primarily involved the collection and analysis of qualitative data (interview, focus group, textual, visual and observational data) across the study sites. Specifically, these wider data sets comprised: interviews and focus groups conducted with residents of the case study developments (to explore their experiences of living in the developments); observational data concerned with how the developments were practised; and secondary (textual and visual) data pertaining to how the developments were formally conceptualized, planned and designed. This ‘intensive’ research design approach [51] was adopted to generate a nuanced, naturalistic and in-depth understanding of research participants’ experiences and perceptions of place. 

In the present paper, we focus on the analysis of a sub-set of data collected for one of the case study developments in the wider study, namely ‘Park Central’—a mixed-use masterplanned development located in Campbelltown (an outer suburb in the southwest of the Sydney Metropolitan Area). In the ‘reactive’ (interview and focus group) data collected in this particular case study site, when asked to reflect on how they perceived and experienced the sustainability of Park Central as a masterplanned development, research participants from across age groups in our sample often framed the ‘sustainability’ of the development in relation to its perceived capacity to accommodate and cater for their (and other residents’) needs across the life course. This emphasis on AIP came to be a recurrent discussion point in the interview and focus group data we collected in Park Central, and a prominent inductive theme [52] throughout our data set for that case study development.

In this paper, we present a thematic analysis of the data collected in Park Central that relates to the ‘friendly residential environments for aging in place with autonomy and independence’ theme of this Special Issue. Specifically, using the thematic analysis steps outlined by Jennifer Attride-Stirling as part of her ‘thematic networks’ approach [53], three key AIP ‘organising themes’ were discerned in the data, and each of those is discussed in turn in Section 3 of this paper.

### 2.1. Park Central

**Figure 1:**
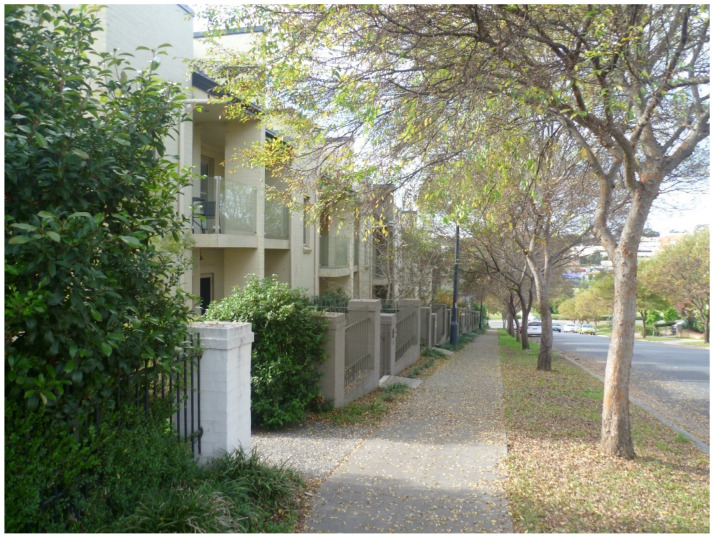
A row of two-storey terraced houses in Park Central. (Photograph by first author).

Park Central is a mixed-use low-to-medium density greenfield masterplanned development occupying 37 hectares of land situated in Campbelltown (a suburb in the Macarthur region of the Sydney Metropolitan Area). In urban design terms, the development has a hybrid (part cul-de-sac/part gridded) street layout, and it primarily comprises a mix of terraced houses (Figure 1) and apartment blocks of two-to-four stories. A summary of the Park Central development compiled by the site developer (Landcom), including an aerial image of the site, is available online [54].

Notably, in terms of the features and form of its urban design, “[t]he NSW [New South Wales] Government…recognised Park Central as setting the standard for future residential development in Sydney’s growth areas” (p. 2, [55]). This was primarily on account of the development being “the first masterplanned mixed-use and medium density development in the Macarthur Region” and it containing “the first studio units, the first apartments for 40 years and the first office development for over 20 years in the area” (p. 6, [56]). More importantly for the present paper, according to the Park Central developer (Landcom) at the time of the delivery of the development, “Park Central embraces the ‘Ageing in Place’ principle of a development mix providing for every stage of life” (p. 12, [56]).

It is important to note at this point that the discursive adoption (by land-use developers), design implementation (by masterplanners and urban designers) and everyday practice (by residents) of planning principles are not one and the same thing [57]. Indeed, the ‘slippage’ between these steps in the development of the wider set of masterplanned environments (including Park Central) studied by the authors is the focus of a separate analysis published elsewhere [44,46]. For the purposes of the present paper, then, it is important to clarify that the predominant focus of the present paper is on residents’ perceptions of and responses to AIP aspects of the built and social environment that were delivered and realised at Park Central.

**Figure 2:**
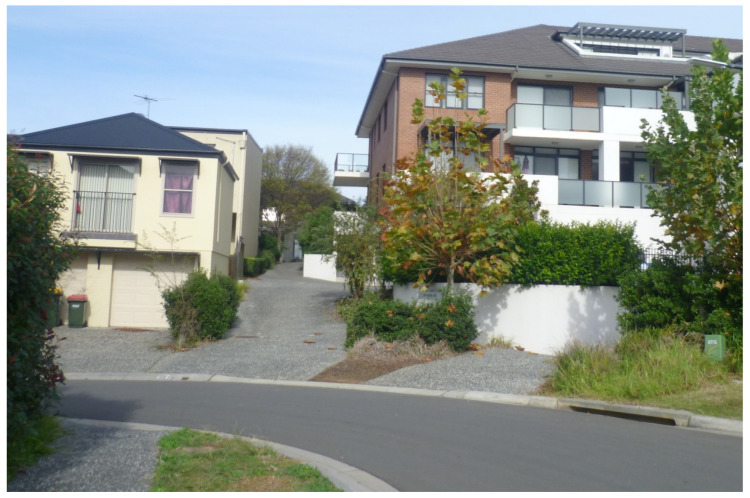
An example of a dedicated footpath (visible leading into the background between the two buildings in the photograph) linking two residential streets in Park Central. (Photograph by first author).

**Figure 3:**
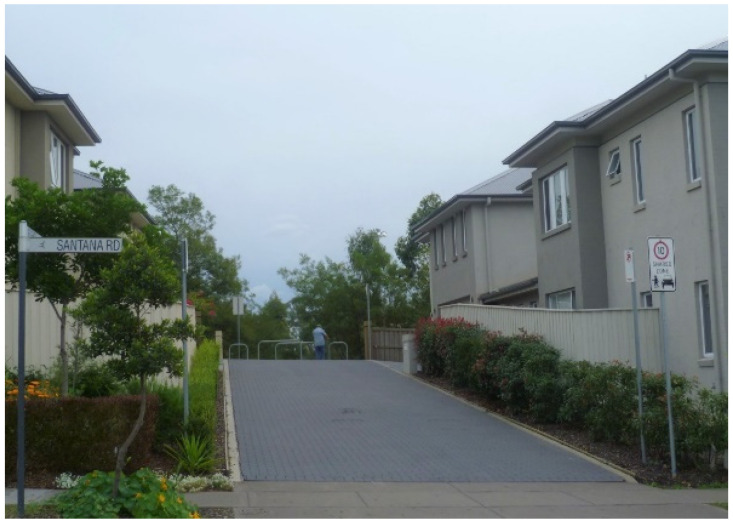
An example of a ‘shared zone’ streetscape in Park Central. (Photograph by first author).

The influence of AIP principles on the masterplanning, delivery and everyday social practice of Park Central could be seen across the features identified by the WHO (pp. 5,6, [6]) as “key aspects of an age-friendly community” (p. 482, [2]). In terms of the masterplanning and urban design of Park Central, the influence of AIP principles to the design aspects of the development are manifest, in particular, in:

- It being situated in close proximity to a range of amenities—including healthcare facilities (esp. Campbelltown Hospital), two very local shopping centres (Macarthur Square and Marketfair Campbelltown), social infrastructure (esp. the Campbelltown Catholic Club) and a public transport hub (esp. Macarthur Railway Station)—identified as important features of age-friendly communities [58];
- The provision, within the development itself, of a “[d]iverse housing mix catering to different lifestyles and age groups” (p. 2, [59]) in order to “retain local residents as their housing needs change” (p. 14, [56]). This dwelling mix comprises apartments, terraced homes, live/work spaces, small lot homes (‘garden homes’), as well as IRT Macarthur (a co-located retirement village and residential aged care centre comprising 262 self-contained independent living apartments in varying configurations and a 60-bed assisted care facility [60]);
- The provision of a range of shared and community facilities within the development itself, including service retail, cafes/restaurants, and the 10.5 ha landscaped open greenspace ‘Marsden Park’ (which, from an age-friendly perspective, is importantly a contained park located in the centre of the development (p. 9, [16]));
- Some attempts within the development itself to create a “walkable community within the context of the ‘car dependant’ south western Sydney region” (p. 14, [56]). These included, for instance, the inclusion of dedicated footpaths linking residential streets in the development (Figure 2), and of some ‘shared space’ residential streets [61] connecting the development to the wider urban area (Figure 3).

In addition, in particular during the early phases of the delivery of the Park Central development, the developer (in coordination with a range of other stakeholders) appointed and resourced (for a fixed-term during the first few years that Park Central was delivered) a ‘Community Development Facilitator’ who welcomed new residents to Park Central, coordinated local community events, helped establish local social networks, and facilitated residents’ participation in the emerging Park Central community. Such support for, and enablement of, opportunities for participation in the civic life of communities, including by social workers [62], has recurrently been identified as a core feature of age-friendly communities (e.g., [5] (pp. 118,119) and [6,11,16]).

### 2.2. Data Collection and Analysis

The findings that follow, therefore, primarily rely on a ‘thematic analysis’ [53] of the reactive (interview and focus group) data collected in Park Central. In our thematic analysis we took a hybrid approach [52], generating themes both deductively (via recourse to the literature) and inductively (out of the accounts of our research participants). In total, our interview and focus group sample comprised 31 residents of Park Central, with 23 residents taking part in interviews and 8 in a focus group. While in this paper we are interested in how adult residents of all ages experience and account for the age-friendliness of Park Central, it is worth noting that 15 of the 31 residents who participated in the interviews or the focus group were aged 65+.

Residents were purposively sampled for variation in a range of demographic (age, gender, ethnicity) and other (type of tenure, car ownership, size and composition of household, years living in Park Central) characteristics. While the demographic characteristics of our sample did broadly reflect the local population demographics, as with all small-n research our aim was not to generate a representative sample of Park Central residents, but rather to ‘sample for range’ and explore a range of adult resident perspectives (p. 13, [63]). Sampling of additional research participants to interview took place until ‘data saturation’ (p. 1897, [64]) was reached, whereby points expressed by new research participants repeated points expressed by research participants who had already been interviewed.

Our interview and focus group data were collected according to a semi-structured approach and using an interview guide. This interview guide was tailored for the focus group discussion, and specifically for the ‘collective conversation’ mode of interaction typical of focus group research [65]. The interview guide covered a range of broad topics, comprising: research participants’ rationales for relocating to Park Central; conceptual understandings of sustainability; sustainable features of the built environment; and sense of community. While both authors of this paper were involved in the conceptualisation of the wider ‘sustainable living practices’ study from which this paper derives, and in the analysis of all of the data generated for that study, all of the Park Central data analysed and/or presented here (interview and focus group transcripts; photographs of the field site; and policy and planning documents) were collected by the first author. These data were collected over a 15-month period in 2012–2013 while the first author was a visiting fellow at UNSW’s City Futures Research Centre.

The discussion of results that follows, therefore, primarily analyses the reactive data collected in Park Central to explore how Park Central residents valued, experienced and shaped Park Central as a locale in relation to conceptual aspects of AIP. All of the interviews were conducted ‘in situ’ in Park Central, either in the research participants’ homes or in a café on site, as was the focus group (which took place in the community hall of the retirement village). This afforded a quasi-ethnographic understanding of how Park Central was experienced, perceived and used, and research participants would often point out or show the interviewer aspects of the development they were describing (e.g., the roads or park visible from the interview location). Where names (of research participants or other people they mention) are given in the analysis that follows, pseudonyms are used.

Finally, to help situate our analysis and contextualise our findings for the reader in the sections that follow, our interview and focus group findings are supported, where appropriate, by photographs taken by the first author on-site at Park Central. Over 250 photographs of Park Central and its surrounding built environment were taken by the author on 10 separate visits to the field site. In addition, over the course of the fieldwork, the first author collected a range of planning and policy documents pertaining to the Park Central development (e.g., masterplan documentation, annual reports from the site developer, sales brochures, etc.), and where appropriate excerpts from these feature in the discussion of our results to help substantiate the claims being made.

## 3. Results

In this section, we discuss three key dimensions of residents’ experiences and perceptions of living in Park Central, before considering these dimensions in relation to the existing literature in Section 4. The three key themes that emerged through our thematic analysis, and that we elaborate on below via recourse to research participants’ responses, are: environmental affordances of age-friendly communities; facilitating age-friendly communities; and the social production of age-friendly communities.

### 3.1. Environmental Affordances of Age-Friendly Communities

Much of the literature on age-friendly cities and communities focusses on aspects of the physical environment that support AIP, including in particular the quality of the built environment and the proximity of local amenities, including healthcare services. These have been conceptualised as environmental ‘determinants’ of active ageing [6] or ‘enablers’ and ‘disablers’ of habits linked to aging-in place [66], though here we conceptualise them as environmental ‘affordances’ [67,68] of age-friendly communities. In this section, we outline the key features (and uses) of the built environment in and around Park Central reported by our research participants as ‘affording’ (or at other times inhibiting) a place for aging.

Across our interview and focus group data, and aligning to the claims of the site developer (Landcom), the close proximity of healthcare services and shops to Park Central, and the location of Marsden Park at its centre, were highly valued features of the masterplanned development. This applied to Park Central residents of all ages in our sample, and when asked why they chose to move to Park Central, residents’ responses routinely emphasised its highly convenient location, as evidenced in the following sample of quotes:

*[W]e’ve got all the medical facilities we need here, three shopping centres in the local area, and a nice place to go and walk if you need to walk*.

*I find it pretty easy [Living in Park Central] actually because it’s all easy access to the shops and everything, everything is close by, shop, train station*. *It’s friendly to have neighbours everywhere and yes, it’s very convenient*.

*Basically, it’s close to everything, close to transport, to the shops, to the public hospital; basically, everything is within walking distance, which is what really appealed to us*.

Notably, these perceived benefits of park Central were contrasted by many residents with their prior experiences of living in other more typically low density and car-dominated suburbs in Sydney, and their expression can be seen as part of a broader move away from the traditional suburban dream in Australia [69].

In short, the development was perceived as comprising (and being very conveniently located in relation to) a range of key local amenities that would serve residents across the life course, including (with the local provision of healthcare services and retirement living) towards the end of life. Thus, residents would report how the close availability of amenities, and in particular healthcare facilities, appealed as they forward-planned for the time that they would no longer be able to drive:

*The other main reason for doing it [moving to Park Central] is if someone can’t drive one day or were injured, we’ve got all the medical facilities we need here, three shopping centres in the local area, and a nice place to go and walk if you need to walk*.

The environmental affordance of active aging did not only apply at the neighbourhood level, however, and residents would also report how the mix and design of dwelling types available at Park Central also afforded active aging. For instance, in one paired-interview the research participants (both of whom were over 65 years-old) reported how they valued that their two-storey terraced home was relatively future-proofed:

*[A] lot of people couldn’t believe that we were buying a house with stairs. But I always said we’ve always got a bedroom downstairs if that’s what we needed, and it’s a big ensuite, a big toilet, shower, basin so you coud take a wheelchair in there if you wanted to*.

Similarly, a couple living in a unit in the retirement village reported how:

*The village units are built, all of them with doors either a metre or very close to a metre wide, to have facility for access by wheelchairs. …In the unit, everywhere is accessible by wheelchair, including the bathroom. The bathroom was built with handrails down the side of the toilet, grab rails in the shower. The shower is big enough to shower three or four people. There is nothing to step over into the shower, you can walk straight in*.

Indeed, when asked about how they perceived the ‘sustainability’ of Park Central, a number of residents reported how for them a key dimension of its sustainability was how the development afforded (through design) AIP. As one interviewee put it:

*Sustainability, from my way of thinking in the sense that …the better the house was built for the older person, the longer the person can stay in there at no additional cost to the government. That’s my way of thinking, a part of sustainability*.

Park Central was by no means perfectly planned for unimpeded inhabitation across the life course, however, and one notable exception to the conduciveness of Park Central to AIP across the life course was the reported absence of high schools within walking distance of the development, which undermined the reported satisfaction with, and suitability of, the area for some of our research participants with school-aged children.

**Figure 4:**
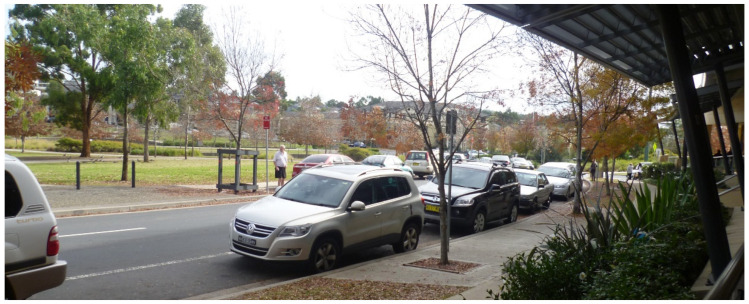
A number of residents reported finding crossing the road to get to/from Marsden Park (in the centre of the Park Central development) difficult on account, in particular, of issues with visibility (caused by cars being tightly parked along both edges of the road, as illustrated in this image) and with the speed of traffic on this particular road (as described later in the paper). (Photograph by first author).

**Figure 5:**
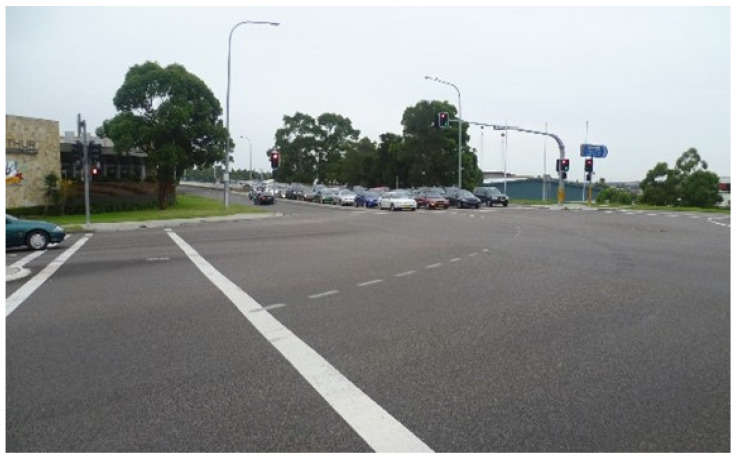
An example of a wide and imposing crossing between the Park Central development and the wider area. (Photograph by first author).

**Figure 6:**
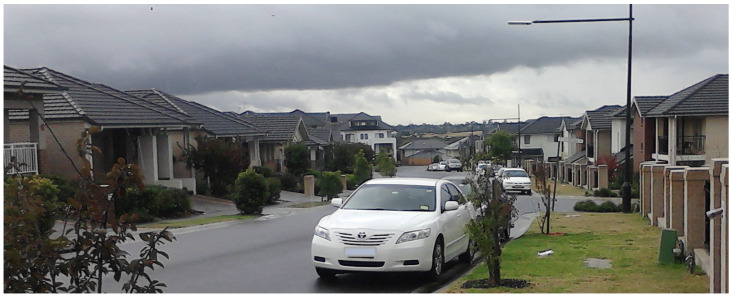
Along certain residential streets in Park Central, such as the one pictured here, a paved sidewalk was only provided along one side of the street. (Photograph by first author).

**Figure 7:**
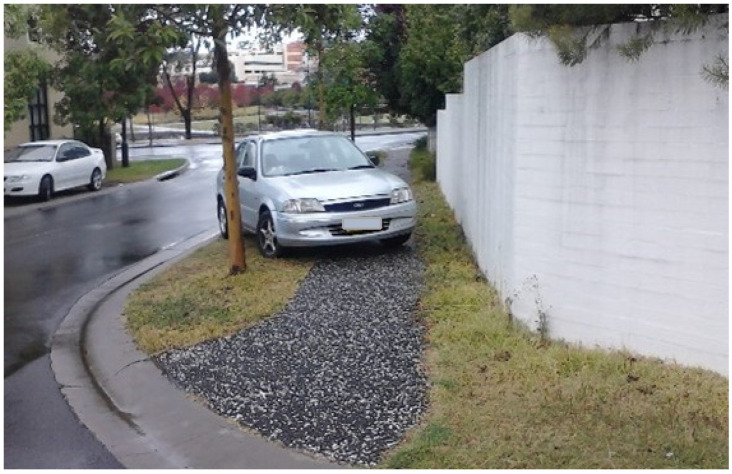
An example of pavement parking in Park Central. (Photograph by first author).

For some, the walkable proximity of two nearby shopping centres (Macarthur Square and Marketfair Campbelltown) was particularly valued, with one interviewee jokingly emphasising this proximity by relaying that “the biggest mistake I made was when I went shopping when we first came in, I’d allow myself 20 minutes to get up town to do shopping, I was back in seven”. For older residents in particular, however, while the local shops were close enough for them to theoretically walk or travel by e-mobility scooter to, this theoretical walkability was undermined by a range of factors, namely:

- the gradient of the streets between Park Central and the shopping centre (with one street referred to as ‘Heartbreak Hill’ on account of its steepness);
- the lack of designated crossings on busy routes within Park Central, and the density and speed of traffic on those routes, some of which had become local ‘rat runs’;
- the lack of visibility for crossing those busy routes (in particular caused by cars parked tightly on both sides of the road (Figure 4));
- a lack of dropped kerbs for crossing roads (for instance when travelling in a wheelchair or by mobility scooter, or with a pram, buggy or shopping trolley);
- those dropped kerbs that are provided being experienced as too steeply inclined to be used without tipping over by motorised wheelchair users;
- the perceived inhospitableness of some the designated crossings over arterial roads that separate Park Central from the local shopping centre (in particular in terms of the width of these crossings (Figure 5) and the short duration of their pedestrian signal phases);
- the lack of pavements on one side of the road in parts of Park Central (Figure 6);
- and regular instances of pavement parking (resulting in blocked walkways) on some streets in Park Central (Figure 7).

Thus, while many of our research participants reported that they did still walk to local amenities, this was often framed as a determined, against-the-odds effort. As one participant put it crossing the busy, heavily parked-up road (Figure 4) running along the edge of Marsden Park (the green space in the middle of the development):

*This is one of the worst [roads to cross] right here, it’s near a hospital and there are sick people going across to the park all the time and they’re slow, but the cars come flying. It’s a bit dicey*.

Crossing this road was described as ‘running the gauntlet’ by one focus group participant, who went on to elaborate that:

*With the private hospital, they need a pedestrian crossing there. People coming from the park area into the private hospital, they’ve got to negotiate the narrow street with the cars moving all the time. …It’s difficult for people to see if they’ve got bad eyesight or if they’re on crutches, or whatever it might be. …You see ladies with prams and two little kids and I’ll tell you, the cars don’t stop for them, and there are a lot of little kids over that new area, you see them in the park. Someone’s going to get skittled*.

Moreover, even where formal pedestrian crossings were provided, where these crossed larger arterial roads running around Park Central (e.g., Figure 5) (and separating it from the nearby shopping centres) some residents experienced these as intimidating and unsafe. For example, when discussing the walk from the retirement village to Market Fair (the closest shopping centre to the village), a focus group participant reported:

*Most of the [traffic] lights [at crossings]…favour the main road. …[N]ot [allowing] sufficient [time to cross] for some people. Especially for some of our residents with walkers and that, because if you walk with them, it is very slow. I wouldn’t like to stop there, but there is another [traffic light] button [on the island] in the middle [of the crossing]*.

Crossings over major roads to get to Macarthur Square on the other side of Park Central were experienced by some non-retirement village residents in a similar way. Thus, one respondent reported how at the crossings to get to Macarthur Square “they should have the traffic lights staggered for the walkers for a little bit longer”. The insufficient time available to navigate these crossings, as well as the intimidating feel of them, was further elaborated by another participant:

*You get halfway across and you’ve got to hurry up. That’s always been a problem around here. …You know what it is, that openness [see Figure 5]. Even these lights and the lights on the corner, you feel like everyone’s looking at you, that it’s odd to walk across*.

In this respect, it is important to avoid framing Park Central, in terms of how it is lived and experienced as age-friendly, as some sort of ‘Gerotopia’ [70]. Having said this, our research revealed that many of the impediments to local walkability and accessibility described above were not insurmountable for, or just accepted as irresolvable by, residents. Rather, as we discuss later in Section 3.3, community-led interventions were developed in an attempt to mitigate or overcome these barriers. These interrelated features of our analysis—community organising and the social production of an age-friendly community at Park Central—are discussed in the following sub-sections. 

### 3.2. Facilitating Age-Friendly Communities

In addition to the importance of how physical features of the built environment are configured and used in relation to age-friendly communities, researchers have also increasingly stressed the importance of ‘relational approaches’ [1] (p. 2) and [57,71]) to studying place. Within this wider focus on “the need to consider physical and social neighbourhood characteristics simultaneously” (p. 2, [1]), a review of age-friendly community models shows that in these models “the trend is to include elements of both the physical and social environment with an ideal of integrating these through appropriate policies, services and structures” (p. 118, [5]). In some of these models (e.g., the University of Calgary’s ‘Elder-friendly community’ model), the emphasis on the social environment includes ‘community development work’ (p. 118, [5]). This resonates with other scholarship advocating for coordinated community development work (including an enhanced and directed role for social services and social workers [5]) in places-for-aging to help “create an environment more friendly to area resident needs” (p. 11, [5]) and to “enrich social capital” through proactive ‘compensation’ for an initial lack of social networks (pp. 58,59, [9]).

In line with such accounts of the importance of attending to both the physical and social environment to achieve age-friendly communities, a recurrent theme in our data was the significant role played by the ‘Community Development Facilitator’. This was someone who was appointed by Landcom and employed by the social service provider UnitingCare Australia (itself an agency of Uniting Church) to introduce new residents to Park Central, foster connections between those residents, and help initiate collective activities there. As with many masterplanned developments, Park Central was delivered in a series of phases, with new cohorts of residents moving into the development as each phase was completed. In this respect, rather than moving into existing and established communities, people were moving, often ‘off plan’, into Park Central as it was being established (as both a physical neighbourhood and a ‘community’).

To facilitate this process, as part of its masterplan Landcom resourced (on a fixed-term basis for the first few years of the site’s phased delivery) the Community Development Facilitator. While much of the role of this appointee was to welcome residents into their new homes (for instance providing them with a ‘welcome pack’ which included information about using appliances installed in new homes on the development), a more significant aspect of the role for many of our research participants was the appointee’s work to initiate and coordinate local community activities that served as a means for residents to get to know one another. As one research participant put it:

*[I]n the early years [of the development], Landcom had a thing going where, in conjunction with Uniting Church, they held various functions for all residents, both in the retirement village and Park Central. And through that, there was interaction, like Mothers’ Day, Fathers’ Day, different functions, going down to Canberra for the Floriade, but that’s now ceased, the funding has stopped*.

In addition to the activities mentioned by our research participant above, the Community Development Facilitator (often working with the nearby Macarthur Centre For Sustainable Living [MCSL]) also:

- distributed, with voluntary assistance from residents, newsletters communicating upcoming community events and activities in and around Park Central;
- organised an open-air movie night in Park Central;
- ran workshops for Park Central residents—e.g., a ‘Going Potty’ workshop (to show residents how they can grow vegetables and small fruit in pots on their balconies) and a Christmas craft-making workshop;
- set-up and coordinated a working group to try and establish a community garden in Park Central;
- helped facilitate and promote a multi-week ‘Living Smart’ course designed to provide participating Park Central residents “with the practical knowledge and skills to take action in their own homes and around the community” (p. 1, [72]). An explicit objective of this course was “[s]upporting and strengthening community relationships” to enable residents “to embrace a connected approach to living, so that long lasting solutions for a quality life now and into the future can be created” (p. 1, [72]).

While the Community Development Facilitator’s activities at Park Central were not always successful—e.g., trying to establish a community garden—a clear externality of these activities was the web of social connections that the activities helped cultivate. Importantly, as evidenced in our interview and focus group data, these activities and the social connections they enabled outlasted the term of the Community Development Facilitator’s appointment, with one retirement village resident even reporting how she personally decided to continue the Facilitator’s ‘meet and greet work’ after the Facilitator’s employment term expired and another reporting that the Facilitator ‘got us going’ in terms of forging social connections in the development. In addition, these activities helped bridge, albeit in a limited way, potential social network boundaries between residents of the aged-care facility and those of the rest of the Park Central development. Thus, some retirement village residents would report having friends in the wider development, with one focus group participant reporting how:

*[T]hey [non-retirement village Park Central residents] do support us when we have things on. When had our White Elephant stall, there was a lot from over there, because they think we’ve got good stuff, see!*

Likewise, an interviewee reported how:

*The things that Sophie [the Community Development Facilitator] arranged, like mother daycare and so on, if it came that they were [taking place] here [in the retirement village] and couples were sitting beside you, they would talk and if the rapport was there and the friendship was…. That’s exactly what happened with us on the bus trip [that Sophie organised]*.

Such links between the retirement village and wider development were further evidenced by the example given in the same interview of a retirement village resident who had rented in Park Central for two years while waiting for a unit in the retirement village to come available.

While our qualitative analysis cannot consider a counterfactual in this regard—i.e., how social networks may have developed had there not been a Community Development Facilitator—for some of our research participants, and in particular for some of our older research participants (both living in the retirement village and the wider development), the role of the Facilitator in this process was reported as being significant. As one retirement village participant put it:

*I think it [the Facilitator’s Community Development work] does help. What she was doing was trying to bring this into a community*.

Elsewhere, a non-retirement village reported how the sustainability and cooking classes organised by the Community Development Facilitator “were fantastic, great. I learned lots of things about recycling”.

At Park Central, then, the masterplanning approach taken included not only conventional urban design interventions oriented (in theory, at least) towards the realisation of sustainable urban form [44], but also a community development intervention (the appointment of a Community Development Facilitator during the initial phases of the delivery and occupation of housing units in Park Central) oriented towards supporting the development of social connections, and thereby social capital, locally. While for many respondents in our sample—and in particular those of working age—the community life of Park Central, and the activities coordinated and initiated by the Community Development Facilitator, were not of interest, for others (and in particular, but not uniquely, for retirement village residents) these activities were an important starting point for connecting with other Park Central residents and for accruing social capital. In the final theme of our analysis that follows we turn to a discussion of uses of these accrued stocks of social capital, and the ways that these uses enabled residents to socially produce the age-friendliness of Park Central.

### 3.3. The Social Production of Age-Friendly Communities

While the role of the Community Development Facilitator at Park Central was contractually short-lived, our data revealed a number of important community development interventions led by Park Central residents that served to enhance the age-friendliness of the development. While it is hard, without an experimental approach, to attribute these interventions directly to the work and influence of the Community Development Facilitator, it was clear in our data that, as per the preceding section, her activities did enable local social connections and get parts of the community going in terms of community organising. This was particularly true for the residents of the IRT Macarthur retirement village situated in Park Central, and in this section we will set out some examples of the ‘social production of Park Central as age-friendly’ in our data set.

As per the discussion in Section 3.2, in the interview and focus group data we collected with residents of the Park Central retirement village we learned that the Community Development Facilitator’s work with those residents inspired them to undertake community organising of their own, and that various facets of the role (e.g., the ‘meet and greet work, but also the organisation of retirement village events including ones open to members of the wider community) were continued by some of the retirement village residents.

Echoing this reported inspiration to continue to proactively organise the community even after the Facilitator had departed, a notable finding in our focus group with residents of the retirement village was that they had self-organised to set up a Residents’ Committee. As one research participant explained:

*[T]he first eight residents in [the retirement village] decided they needed to coordinate. They set up what they called a coordinating committee or some such thing. By the time we had 60 or 70 people in there [the retirement village], and that was only a matter of three months and that’s when we came in, they decided they needed to formalise it. We had a meeting of those 60 or 70 people and they agreed to have a Residents’ Committee. They elected eight people to be the Residents’ Committee and that committee set up a constitution … So then we had a Residents’ Committee who had a chairperson, secretary and treasurer and six other members and they’re elected annually*.

Notably, as this research participant pointed out, this organisational model differed starkly from “IRT’s system in the previous 31 areas in which it operated [whereby]… it appointed or the residents appointed someone called a Resident Councillor and that was a single person”. This Resident Councillor was responsible for “liaising with the operator and for being an advisor to all the residents and helping them to sort out their problems, either with other residents or with the operator”. At IRT Macarthur, the Residents Committee did not replace the Resident Councillor, but rather supplemented it (with the Resident Councillor being invited to sit on the Committee). A key purpose of the Committee, then, was to help resource and organise social activities (such as a craft group, a men’s group, a games group, fundraising activities and a monthly ‘sausage sizzle’) at the retirement village. In addition, the Committee worked to coordinate adjustments to the retirement village infrastructure to improve its functionality (e.g., installing a can crusher in the village’s garbage area to improve recycling practice there and instituting a common apartment door signage system residents could use to signpost to others whether they were at home or not). As the research participant quoted above put it, “right from the word go, right from the first Residents’ Committee, we knew that we had to get people to develop a spirit of cooperation and community”.

Beyond the inception of the Residents’ Committee, and the activities and interventions the Committee delivered, a number of other examples of this ‘spirit of cooperation and community’ at the retirement village were evident in our data set. These included:

- Suitably qualified retirement village residents organising and operating, voluntarily, a weekly bus service from the retirement village to the local shopping centres, thereby enabling residents whose access to those shops was constrained or impeded (see Section 3.1) to circumscribe some of those constraints or impediments. This service was enabled by IRT conditionally permitting self-care residents to use the retirement village bus, though this required residents to volunteer as drivers for any social trips they wanted to organise. In addition to the weekly shopping service, residents also organised and operated other regular social outings using the IRT-supplied bus.
- Residents petitioning, albeit unsuccessfully, for the provision of a ‘high care’ unit in the vicinity of the retirement village.
- Residents working for local community organisations in the wider local area and sitting on local area committees—e.g., one research participant reported how she volunteered with the local RSL club and chaired the local Senior Issues Group which reports to Campbelltown City Council.
- The organisation of 3–4 ‘block parties’ a year to enable residents on each block to socialise with one another.
- A group of residents organising a group visit to a former resident who had recently been moved to a high care facility that was not local to the retirement village.

These community-minded activities can be seen to enhance the age-friendliness of the retirement village (and its wider setting) for retirement village residents. For instance, ‘accessible transportation’, such as the bus service enabled by volunteer drivers from the retirement village, has been identified elsewhere “as a key feature that supported aging-in-place” (p. 155, [66]). More broadly, though, these activities can be conceptualised as processes through which the retirement village and its hinterlands were socially produced as age-friendly. This conceptualisation draws on Henri Lefebvre’s [73] theories of the production of space, and of the socio-spatial dialectic (whereby the production of social space is a product of the dialectical relationship between spatial structures—the built environment—and social practices).

**Figure 8:**
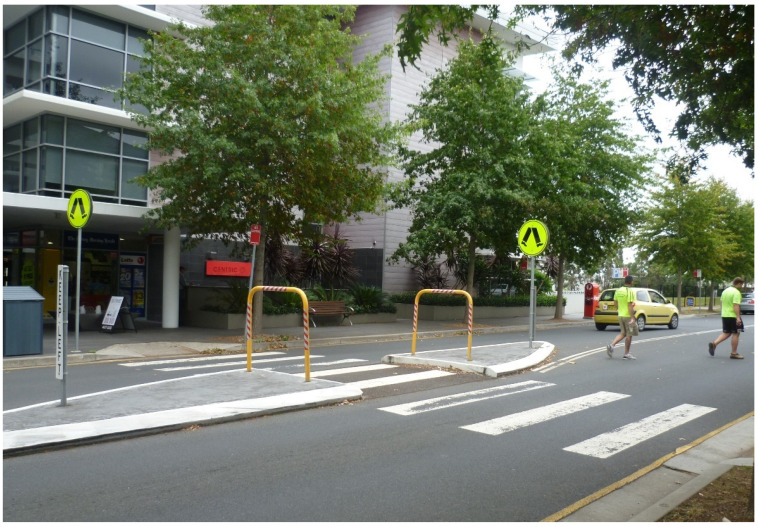
The pedestrian crossing over Hyde Road that IRT Macarthur retirement village residents successfully lobbied to be installed. (Photograph by first author).

Perhaps the most dramatic example from our dataset of this ‘social production’ of (age-friendly) space at play relates to retirement village residents successfully organising, over a three-year period, to demand the provision of a pedestrian crossing (Figure 8) over the main road from which the retirement village is accessed.

Thus, residents reported how access to the newsagents (just visible on the left-hand side of Figure 8) and healthcare services (among other things) on the other side of the road from the retirement village was historically severely impeded by the lack of a pedestrian crossing. In response to this barrier to active aging, a group of residents campaigned both through formal avenues—letter-writing and talking to Aldermen—and direct action to have a pedestrian crossing installed. As one research participant put it, they made “a phone call to a reporter, and…their photographer, organising a group of people from the hospital, walking frames, sticks, walking across there, about 20 of them” to pose next to the site of the demanded crossing for a local newspaper report. On the back of this reporting, another research participant reported how:

*A Councillor came to look at it one day to see how bad it was and […] an engineer from the Council, and I arranged for it to be very busy that day. But eventually it [the crossing] went in and it’s been used every day*.

**Figure 9:**
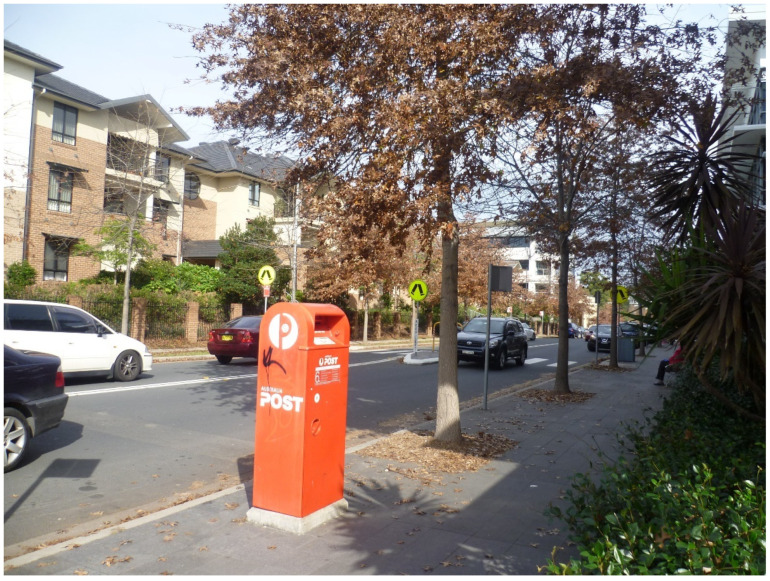
A post box installed on Hyde Road (as a result of retirement village resident campaigning) and situated near the retirement village and pedestrian crossing discussed above (both visible in the background of the photo). (Photograph by first author).

In addition to lobbying successfully for this crossing, the retirement village residents also successfully campaigned for a post box (Figure 9) to be installed near to the entrance of the retirement village and to the pedestrian crossing discussed above.

Through the examples discussed in this section, then, we argue that the ‘age-friendliness’ of Park Central is not just a function of its urban design and spatial structures, but rather that it is produced dialectically through the relationship between spatial structures and social relationships. Importantly, the particular components of the dialectical production of space observed at Park Central are not generalisable to other masterplanned settings. However, we do contend that the findings we set out above (in particular in Section 3.1 and Section 3.2) may be ‘transferable’ [74] to other masterplanned neighbourhoods, and that based on our evidence the social production of age-friendly space may, conceptually, be facilitated in settings where social interventions designed to foster social connections and community development initiatives are implemented.

## 4. Conclusions

As models of, and urban design interventions for, age-friendly communities have become increasingly prevalent, an increasing number of calls for evidence about how such communities are experienced and how successful they are (visàvis a range of outcomes) have been made. To date, the availability of such evidence (at the neighbourhood level) has been limited. As Sánchez-González et al. have put it, “[t]he academic literature contains little information regarding the interventions that create age-friendly cities and communities in order to promote active ageing” (p. 1, [18]). Studying masterplanned developments conceived and developed with the AIP principle in mind offers a unique opportunity to study purposefully age-friendly communities, and in this paper we set out an analysis of how residents of one such development (Park Central, in Australia) experience and perceive it as a ‘place for aging’. The value of this contribution is informed by the observation made by others that “[d]etailed investigations of AIP in situ provide more detailed accounts of the lived experience of older adults in ways that ‘generic treatment’ of neighborhood may not” (p. 7, [1]). Moreover, as has been argued elsewhere in relation to the value of inductive designs in evaluation [75], we argue that such detailed investigations help elucidate the potential “mechanisms by which environments affect health” (p. 16, [29])—a facet of AIP research that has been assessed to be underdeveloped to date [29].

In line with the wider literature on age-friendly cities and communities, in our data, we found that Park Central residents put a lot of emphasis on the quality of the physical environment as a determinant of the age-friendliness of the development. Notably, it was clear that in many ways the built environment in-and-around Park Central (and its use by car and other vehicle drivers) was not conducive to an age-friendly community. This was in part a function of slippages between the principles and implementation of masterplanning at the site [44], but also seemed to reflect how urban design intentions and uses of the built environment do not always align [76]. For instance, pavement parking on the site (which inhibited walkability) was not a design intention, although it was reported by research participants to be a function of the appeal of Park Central as what has come to be termed a ‘20 min neighbourhood’ [77] with a wide range of amenities in close proximity. Here, then, we see one dimension of an age-friendly community (availability of local amenities) serving to undermine another dimension (walkability).

Stepping back from our close analysis of research participants’ experiences of the local environment and its accessibility at Park Central, then, one finding of our research is that even when residential settings have been purposefully designed for age-friendliness, negative externalities and unwanted outcomes may transpire. In short, ‘age-friendliness’ cannot just be solved through carefully planned and well-intended urban planning or design solutions, or through focussing “narrowly on technical or architectural guidelines or design specifications” (p. 118, [5]) as some senior-friendly programs have been found to do.

Here, then, the second theme in our preceding analysis is important. Namely that the age-friendliness of new pieces of residential urban fabric can be enhanced through interventions in the social as well as the physical environment. In the case of Park Central, then, for some of our research participants, the role of the (albeit temporary) Community Development Facilitator in helping foster social connections was invaluable. Critically, the work of the Facilitator impinged on how Park Central was perceived as an age-friendly community not only directly through the social connectivity it enabled, but also indirectly through the use of stocks of social capital that those social connections helped generate and unlock.

This ‘use of social capital’ took the form, for instance, of some local residents’ (and in particular older residents’, who self-reported as being relatively time-rich) successfully campaigning, for instance, for the installation of a pedestrian crossing between the aged-care facility and local amenities on the other side of a busy road running alongside it. In doing so, residents were able to address some of the age-friendly shortcomings of the built environment at Park Central discussed in Section 3.1, and to thereby ‘socially produce’ the development as more age-friendly for themselves. Crucially, however, because Park Central was masterplanned in such a way that the retirement village was integrated into the wider development (rather than developed as an isolated and self-contained facility), such social productions of space benefitted not only the retirement village residents (p. 59, [9]) but also the wider community of users of the neighbourhood (e.g., in Figure 8 we can see the pedestrian crossing being used, albeit casually, by ‘tradies’ [or tradespeople] working in the area). In this example, then, it is the intersection of physical (urban design) and social (community development) features of the masterplanning process at Park Central that help enable residents to shape the age-friendliness of their community. This can be conceptualised as a socio-spatial dialectic, whereby interventions in the physical and social environment of Park Central (p. 118, [5]) are in a dynamic relationship that is mutually constitutive.

Interventions in the social environment to cultivate community organising have been critiqued elsewhere for failing to adequately address “individual differences and values in modern communities that have become increasingly diverse and complex” (p. 118, [5]). This is an important observation, and indeed the community facilitation work undertaken at Park Central can be seen to have suffered social inclusion issues, with many of our research participants reporting being unaware of, or disinterested in, the work of the Community Development Facilitator. However, despite this unevenness of the reach of the activities led by the Community Development Facilitator, we contend that the community organising conducted by those who were more engaged resulted in changes to the local environment (such as the installation of a pedestrian crossing and of a post box) that benefitted all Park Central residents, regardless of their levels of engagement with organised community activities. This is not to say that efforts should not be made to diversify community development work in age-friendly communities, and thereby to maximise social inclusion. Rather, it is to flag that even for community members who are too time-poor to participate in these activities (as was the case for a significant number of our interviewees, including some who worked two jobs or who had childcare commitments) or who just did not want to be involved in them (e.g., because they prioritised family commitments), material benefits to them from others’ (such as older residents, some of who are relatively time- and skills-rich) engagement in those activities can accrue.

In addition, it has been observed that much of the age-friendly community literature:

*is essentially descriptive. It provides considerable detail about…a range of approaches to fostering age-friendly communities—in terms of models of governance—and a range of outcomes regarded as age-friendly in terms of both physical and social characteristics. However, there has been limited documentation of the effectiveness of specific approaches or evaluation of the impact of specific processes or outcomes on older people’s lives*.

(p. 118, [5])

Owing to the level of detail afforded by our intensive research approach, we were able to identify a few important shortcomings of the community facilitation approach taken at Park Central that, while not providing a comprehensive evaluation, speak to the effectiveness of the approach and indicate ways that community facilitation work could be enhanced in other settings. These shortcomings comprise:

- The observation that community activities were often scheduled during work hours, thereby systematically excluding people of working age. Should a community facilitation approach be adopted elsewhere, therefore, care should be taken about when events are scheduled to take place, as well as who they are organised for.
- A concern (reported by one research participant) about the Community Development Facilitator working for an agency of a religious organisation (Uniting Church Australia). For the research participant, such an affiliation is “going to exclude people straight away”.
- A communication strategy—which primarily comprised distributing newsletters to residents setting out upcoming community events—that was not always effective, with one research participant who was unaware of the activities organised by the Community Development Facilitator describing how “junk mail…just goes straight in the bin”.
- The Community Development Facilitator being unable to secure from the developer a piece of enclosable land on which to plant a community garden (a type of intervention that has been shown to have numerous significant active aging benefits [78]).

With these shortcomings in mind, it is important more broadly to note that age-friendly interventions in the social environment of places-for-aging need to (i) be developed with a close understanding of the local socio-cultural context in order to maximise the reach of those interventions and (ii) have the in-principle support of the land owner/developer for community-led interventions in the physical environment (such as a community garden).

As Lewis and Buffel (p. 7, [1]) assert, places of aging are ‘dynamic’, and “the person-environment fit is not static”. Such dynamism implies, if there was any doubt, that even with the best intentions and closest adherence to principles of AIP, age-friendly communities cannot be realised through urban design/physical environment interventions alone. Rather, the dialectical socio-spatial production of age-friendly space is required—a process that can dynamically shape and enhance the age-friendliness of a given setting. This process relies on interventions in the social environment of neighbourhoods designed to facilitate social connections, cultivate social capital and get community development activities going. Even where such interventions are not continuous, and do not reach all residents of a neighbourhood, our findings show that they can still result in neighbourhood liveability benefits not only for those who engaged with the interventions but also for the wider community (p. 48, [79]). In the case of Park Central, this points to an important (but perhaps often overlooked) civic role performed by ‘retired not expired’ [80] older community members able and willing to participate in community organising, who not only enhanced the age-friendliness but also (as a ‘spillover benefit’ [81]) the broader liveability of the development.

## Data Availability

The data presented in this study are not publicly available due to the conditions agreed with research participants regarding data collection when informed consent was obtained.
